# Fracture Toughness and Elastic Modulus of Epoxy-Based Nanocomposites with Dopamine-Modified Nano-Fillers

**DOI:** 10.3390/ma10070776

**Published:** 2017-07-10

**Authors:** Kwang Liang Koh, Xianbai Ji, Aravind Dasari, Xuehong Lu, Soo Khim Lau, Zhong Chen

**Affiliations:** 1 School of Materials Science and Engineering, Nanyang Technological University, 50 Nanyang Avenue, Singapore 639798, Singapore; edwinkohkl@gmail.com (K.L.K.); XJI005@e.ntu.edu.sg (X.J.); ASXHLu@ntu.edu.sg (X.L.); 2 Singapore Institute of Manufacturing Technology, 73 Nanyang Drive, Singapore 638075, Singapore; lausk@SIMTech.a-star.edu.sg

**Keywords:** polydopamine, montmorillonite clay, carbon nanofibre, fracture toughness, elastic modulus

## Abstract

This paper examines the effect of surface treatment and filler shape factor on the fracture toughness and elastic modulus of epoxy-based nanocomposite. Two forms of nanofillers, polydopamine-coated montmorillonite clay (D-clay) and polydopamine-coated carbon nanofibres (D-CNF) were investigated. It was found that Young’s modulus increases with increasing D-clay and D-CNF loading. However, the fracture toughness decreases with increased D-clay loading but increases with increased D-CNF loading. Explanations have been provided with the aid of fractographic analysis using electron microscope observations of the crack-filler interactions. Fractographic analysis suggests that although polydopamine provides a strong adhesion between the fillers and the matrix, leading to enhanced elastic stiffness, the enhancement prohibits energy release via secondary cracking, resulting in a decrease in fracture toughness. In contrast, 1D fibre is effective in increasing the energy dissipation during fracture through crack deflection, fibre debonding, fibre break, and pull-out.

## 1. Introduction

Epoxy has been used extensively for engineering applications due to its combined advantages of high stiffness, strength, creep resistance, and chemical resistance. However, its fracture resistance is relatively low because of the rigid network of cross-linked polymer chains. Significant effort has been made in the past to improve various properties of epoxy, such as changing the backbone chemistries of the epoxy and curing agent [1], modifying the thermoset resins with thermoplastic polymer [2,3,4], and creating a multiphase polymeric system by mixing dispersed elastomeric and thermoplastic phases with the epoxy. Nevertheless, these explored methods have only provided limited improvement to the fracture toughness of the highly cross-linked, high glass transition temperature epoxy and their composites. Challenges remain when epoxy materials are introduced to applications in the aerospace or automotive industries [5,6].

Towards the end of the 20th century, the concept of nanocomposites started to emerge, offering the industry with promising results and a unique level of property improvement. This method involves the incorporation of nano-sized organic and inorganic nanoparticles to the epoxy matrix. Researchers from Toyota [7] increased the thermal and mechanical properties of polyamide-6 nanocomposite by adding a very small amount of clay nanoparticles (~4 wt %). Subsequent studies by other researchers also reported significant improvements in stiffness and strength by adding nano-sized clay fillers compared to micro-size in the same polymer clay, at low loading percentages of about 1–5% by weight [8,9,10,11]. Other forms of nanofillers including spherical particles, nanotubes, nanofibres, and other 2D materials have been investigated, showing great improvement in the mechanical properties of the materials [12,13,14,15].

Generally, surface treatment of nanofillers is required for better interaction with hydrophobic polymer matrices. Mussel adhesive proteins (MAPs) have received a great amount of attention for their capability to adhere effectively to different types of surfaces. MAP contains a large quality of a particular amino acid known as dopamine (DOPA) that is responsible for the strong adhesion of mussels on different types of marine surfaces [16]. Podsiadlo et al. [17] have applied a small amount of DOPA to artificial nacre to achieve a composite film. Due to its strong adhesion, DOPA acts as an effective load transfer agent and, thus, improves the overall stiffness and toughness of polymer composites. Han et al. [18] applied DOPA-intercalated clay nanosheets for the enhancement of both adhesive strength and toughness of hydrogel for skin regeneration. Zhu et al. [19] added DOPA-coated carbon nanotubes into poly(vinylidene fluoride) matrix, the resulting composite was able to achieve a balanced high permittivity and low dielectric loss. DOPA-coated fillers have also been reported for enhancing thermal stability, mechanical strength, and tribological performance of nanocomposites [20,21,22].

Despite these efforts, there have been very limited studies on the fundamental root causes that contribute to the cracking process, i.e., the micro-mechanisms of a fracture in the nanocomposite, especially the filler and filler-matrix interaction. In this study, we investigated how DOPA coating treatment affects the fracture toughness and elastic modulus of epoxy-based nanocomposites. Two types of nanofillers were used: montmorillonite (MMT) clay as a 2D filler, and carbon nanofibre (CNF) as a 1D filler. Composites with and without DOPA coating on the fillers were tested and analysed. The comparison between the 1D and 2D fillers enables us to understand how the form factor affects the fracture toughness and fracture process.

## 2. Results and Discussion

### 2.1. Verifications of DOPA Presence on D-Clay

**Figure 1:**
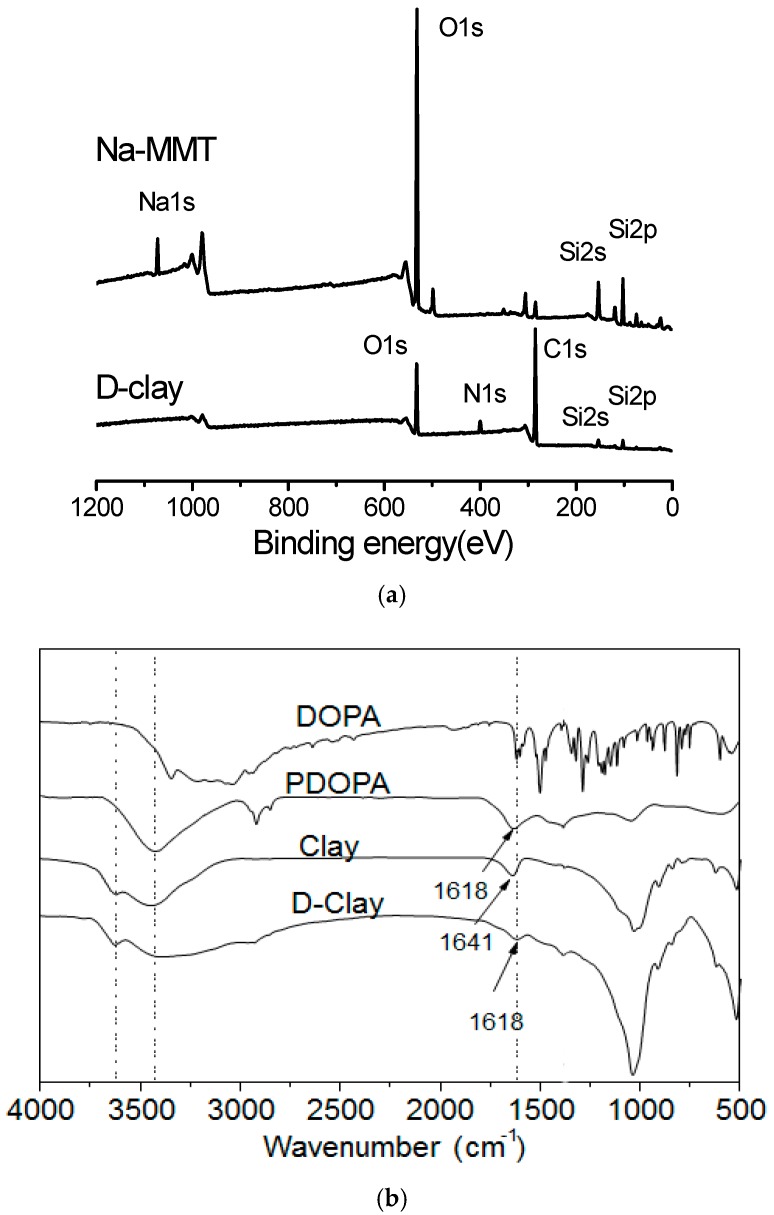
(**a**) XPS spectra of MMT clay and D-clay; and (**b**) FTIR spectra of MMT clay, D-clay, DOPA and PDOPA.

The DOPA-coated clay (D-clay) particles were examined using X-ray photoelectron spectroscopy (XPS) to verify the presence of DOPA on the MMT surface. The results from XPS revealed that the sodium atoms were no longer detected and the signals for oxygen and silicon were largely reduced as compared to the untreated MMT (Figure 1a). At the same time, a higher concentration of carbon and nitrogen were detected on the D-clay surface similar to the observations reported on surface functionalized nanofibres in [23]. Furthermore, using Fourier transform infrared spectroscopy (FTIR), similar bands can be found for both polymerized dopamine (PDOPA) and DOPA (Figure 1b). Thus, we conclude that DOPA was polymerized on the surface of the MMT. It is well-known that the intensive band in MMT at 1015 cm^−1^ is due to in-plane stretching of Si–O, and the out-of-plane stretching will appear as a tiny shoulder at 1115 cm^−1^. Additionally, bending vibrations of the hydroxyl groups of water generally occur at 1641 cm^−1^ in MMT (stretching will be at ~3430 cm^−1^). The relatively broad band between 3200 and 3500 cm^−1^ that appeared in D-Clay compared to MMT is a combination of stretching vibrations of –NH and –OH. The band at 1618 cm^−1^ in D-Clay assigned to C=C further confirms the presence of dopamine.

**Figure 2:**
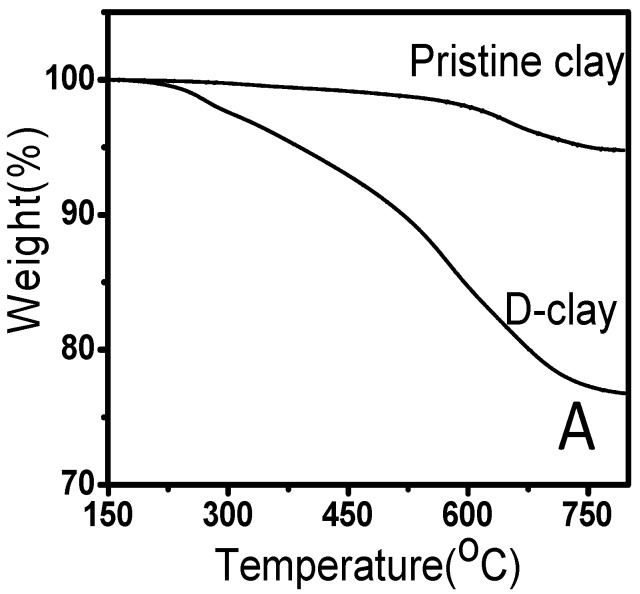
TGA curves of pristine clay (MMT) and D-clay (1.5 mg/mL DOPA concentration) in air.

Thermogravimetric analysis (TGA) was used to determine the amount of DOPA polymerized on the surface of the MMT. From the TGA curve shown in Figure 2, the degradation of D-clay begins at ~230 °C and ends at about 700 °C. The weight loss between 600 °C and 800 °C is caused by dehydroxylation of MMT clay which suggests the loss of structural –OH groups. From this result, we can conclude that the PDOPA has relatively low thermal stability, therefore, the amount of PDOPA must be controlled to achieve the expected adhesive interaction while maintaining the thermal stability required for the composite. A series of tests was conducted with DOPA loading from 0.1 to 2.0 mg/mL, and the thermal stability was determined by the starting temperature of degradation. It was found (results not shown here) that 1.5 mg/mL was able to provide the most stable DOPA coating, as well as good dispersion of the fillers for the desirable mechanical properties. Higher concentrations would not increase the thermal stability and coating thickness any further. Therefore, unless specified, all the results reported will be based on 1.5 mg/mL DOPA concentration.

**Figure 3:**
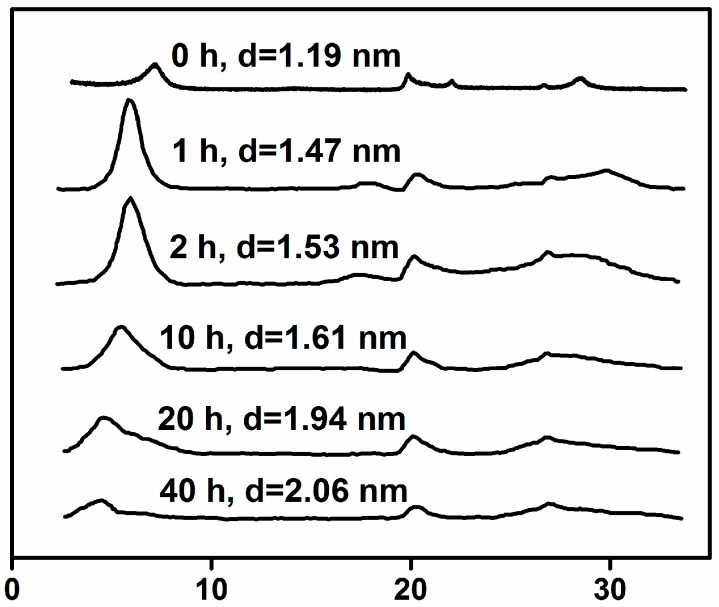
X-ray diffraction (XRD) curves of D-clay obtained at different polymerization times.

The D-spacing of the samples were measured using X-ray diffraction (XRD) and results are shown in Figure 3. Typical d-spacing of the MMT, as-received, was found to be around 1.19 nm. During the polymerization of the DOPA in the presence of MMT, the d-spacing between different clay layers increases, suggesting the intercalation of DOPA chains. Different polymerization times were used to obtain the optimum time. After polymerization for 1 h, the D-clay exhibited a peak at 2θ = 6.02°, which directly corresponds to a d-spacing of 1.47 nm using Bragg’s law (an increase of ~0.28 nm from pristine MMT). Considering the polymerization times and the achieved d-spacing, a 2 h polymerization time is chosen as the optimum condition. 

### 2.2. Filler Dispersion Characterisation of Nanocomposites

**Figure 4:**
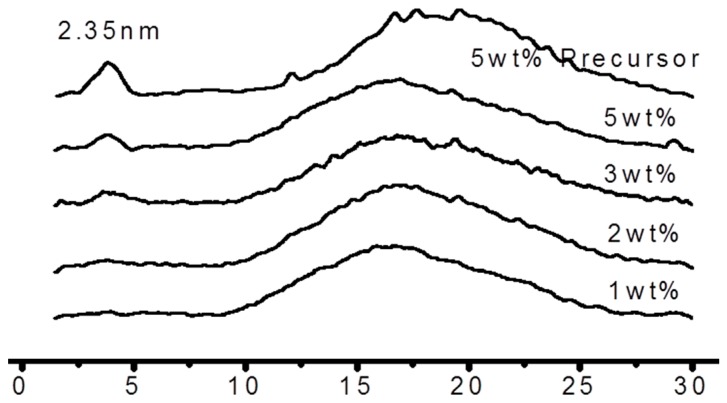
XRD curves of D-clay epoxy at different weight loadings.

Figure 4 shows the XRD scan of composites with different D-clay loading. A reference of uncured sample without the addition of the amine hardener (labelled as 5 wt % precursor) was also plotted. The d-spacing of the D-clay has expanded from the initial 1.53 nm to 2.35 nm after epoxy resin was added into the solution. This signifies that the epoxy resin has intercalated into the D-clay layers.

**Figure 5:**
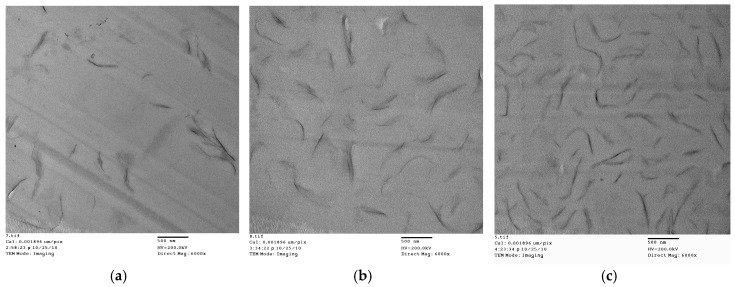
TEM micrographs of the D-clay epoxy at different weight loading. (**a**) 1 wt %; (**b**) 2 wt %; and (**c**) 5 wt %. The scale bar represents 500 nm in all figures.

After the epoxy resin was cured with the amine hardener, the peak at 2θ = 3.8° weakened significantly. This result shows that during curing, intercalation of D-clay has taken place. This extent of intercalation could lead to great improvement in both mechanical and physical properties of the polymer clay composite [24,25,26,27]. Transmission electron microscope (TEM) images taken from the cured D-clay epoxy nanocomposites show that the D-clay are in the form of thin tactoids formed by a few layers and randomly distributed throughout the epoxy matrix (Figure 5). The thickness of such tactoids is about 10 nm and the length to thickness ratio in the range of around 50. At the same time, completely exfoliated single layers can also be observed in the area near the tactoids. It is, therefore, concluded that the PDOPA coating on the MMT greatly benefits the intercalation and exfoliation of D-clay within the epoxy matrix because of the strong interaction between PDOPA and epoxy matrix to give a higher magnitude of intercalation and partial exfoliation of the D-clay within the epoxy matrix.

**Figure 6:**
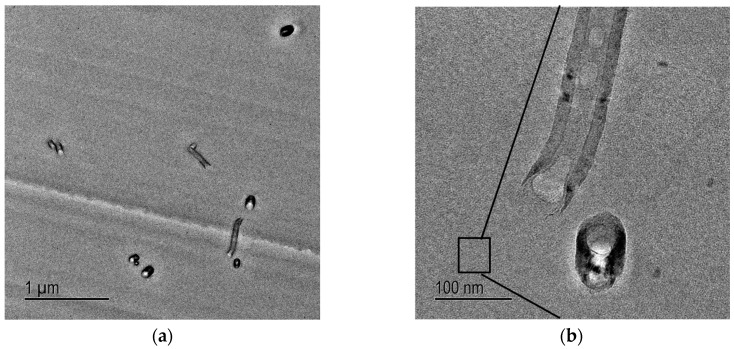
TEM micrographs of the D-CNF epoxy nanocomposites with (**a**) low and (**b**) high magnifications.

On the other hand, TEM images taken from the cured DOPA-coated carbon nanofibre (D-CNF) epoxy nanocomposites show that the D-CNF are individually and randomly dispersed throughout the epoxy matrix (Figure 6). PDOPA coating on the CNF ensures good dispersion of D-CNF within the epoxy matrix, and this is attributed to the strong interaction between PDOPA and the epoxy matrix.

### 2.3. Storage Modulus

**Figure 7:**
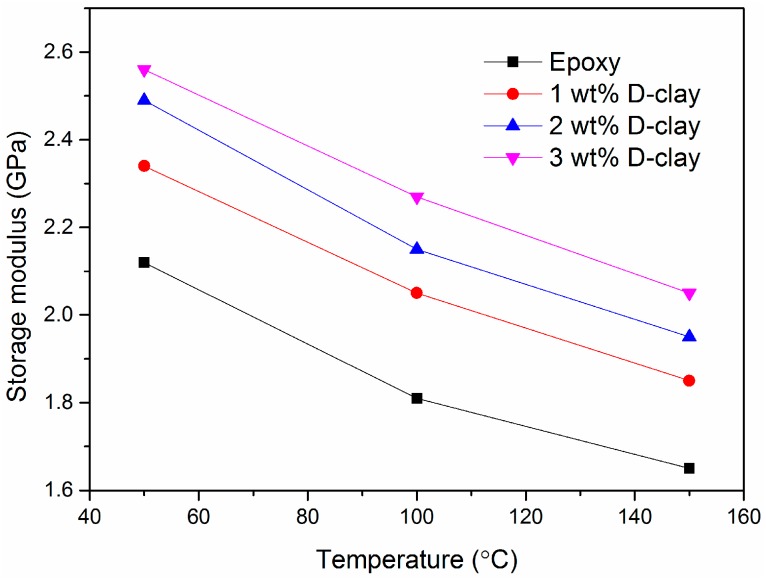
Storage modulus of D-clay epoxy at different weight loading, measured by dynamic mechanical analysis (DMA).

As shown by the dynamic mechanical analysis in Figure 7, by adding D-clay into the epoxy matrix, the storage modulus has an impressive improvement over epoxy without any filler addition. The increase in modulus is close to 30% at 3 wt % clay loading. Among different loadings (0–3 wt %), the 1 wt % loading seems to have relatively better improvement compared to neat epoxy than the subsequent increase of the loading. We deduce that at lower clay loading, the clay is able to disperse and distribute better within the matrix.

**Figure 8:**
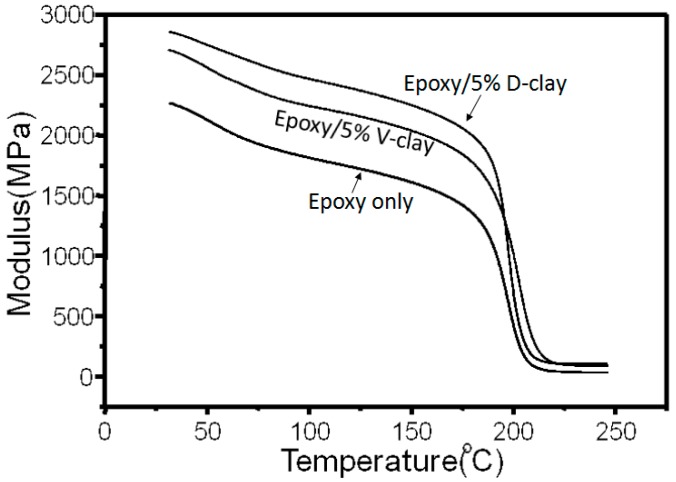
DMA curves of neat epoxy, epoxy with 5 wt % D-clay, 5 wt % V-clay, and 5 wt % organoclay.

Figure 8 shows that the storage modulus of D-clay composite is higher than that of untreated virgin clay (V-clay) composite, while the epoxy without filler is even lower than the V-clay composite. The comparison indicates that the interfacial interactions, and thereby storage moduli, are indeed enhanced by the DOPA treatment.

### 2.4. Young’s Modulus

**Figure 9:**
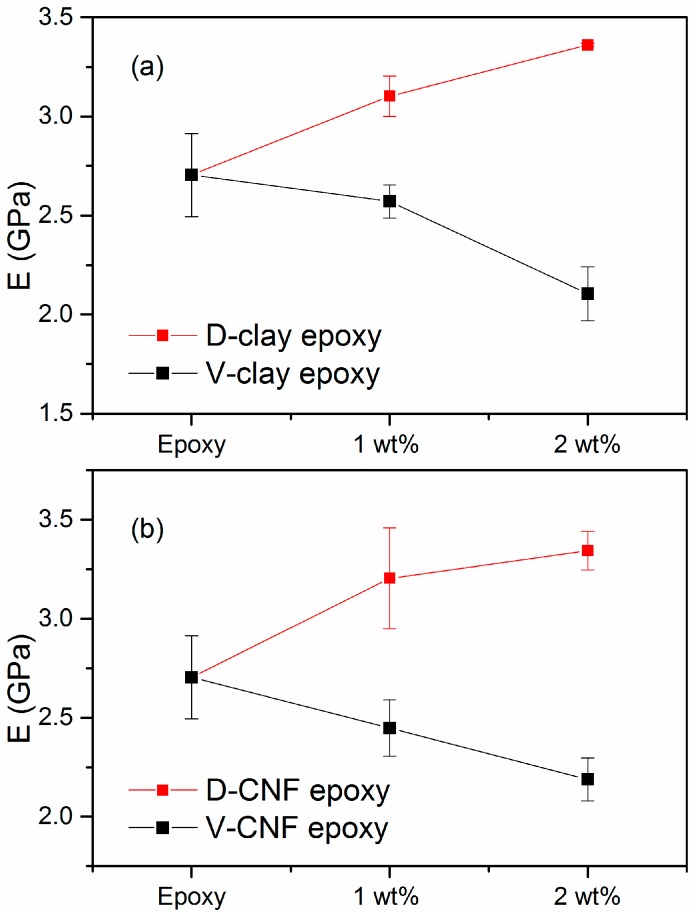
Young’s modulus of composites with different filler loading (**a**) MMT clay (**b**) CNF.

As shown in Figure 9a, there is an increase of ~13% in Young’s modulus of the composites with 1 wt % addition of D-clay. At 2 wt %, the increase is ~25%. From previous XRD results and TEM observations, D-clays are intercalated within the epoxy matrix with some of the clay layer completely exfoliated in the epoxy matrix. This illustrates that this state of dispersion helps in improving the stiffness of D-clay epoxy nanocomposites. In contrast, the Young’s modulus of epoxy V-clay composites dropped by about 24% with addition of 2 wt % V-clay. 

Similarly, as shown in Figure 9b, the Young’s modulus of D-CNF composite increases with filler loading. There is an increase of ~18% in Young’s modulus of the composites with 1 wt % addition of D-CNF. At 2 wt %, the increase is ~ 24%. The Young’s modulus of the untreated virgin CNF (V-CNF) composites dropped by about 19% with the addition of 2 wt % V-CNF.

The contrast between DOPA-coated fillers and virgin fillers clearly indicates the importance of interfacial adhesion between the matrix and the fillers. The D-clay and D-CNF, having a strong adhesion with the matrix, are able to enable effectively transfer stress from the epoxy matrix to the filler (which has a higher stiffness). As a result, the overall elastic constant increases because the filler is able to undertake some of the stress. Untreated filler, on the other hand, does not have the strong interface with the matrix. Therefore, the stress is largely undertaken by the matrix only. The Young’s modulus decreases because of loss of volumetric percentage of the load bearing phase of the epoxy matrix.

### 2.5. Fracture Toughness

**Figure 10:**
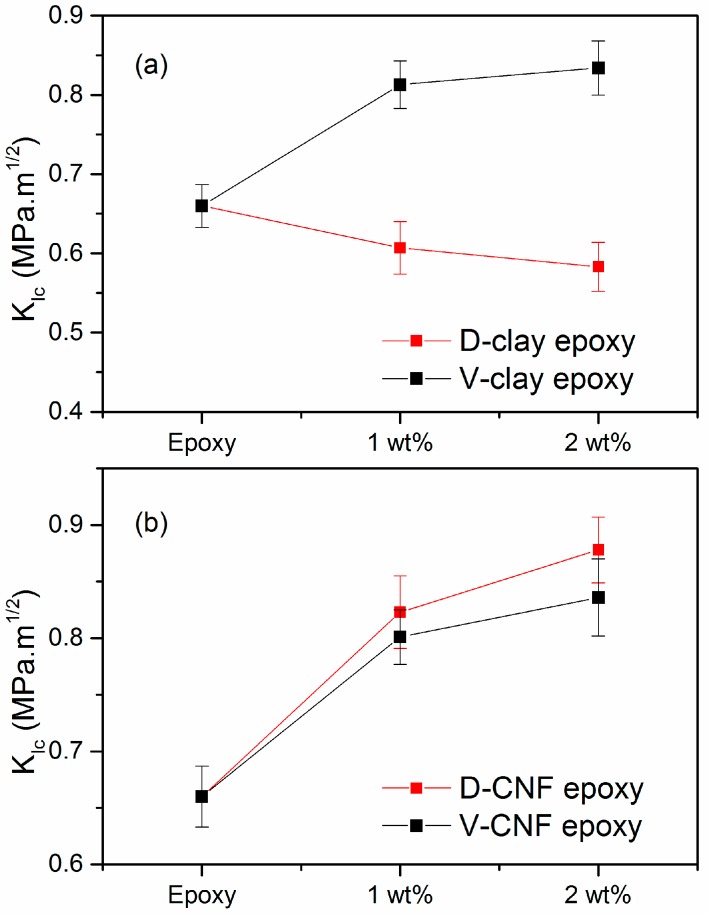
Critical stress intensity factor of nanocomposites with different filler loading (**a**) MMT clay (**b**) CNF.

**Figure 11:**
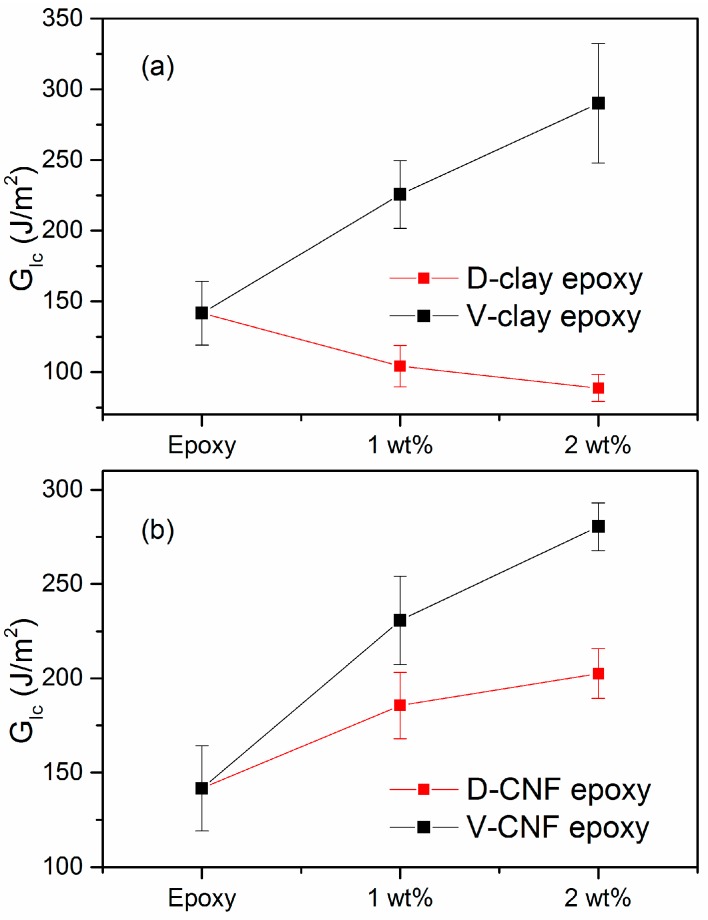
Critical strain energy release rate of nanocomposites with different filler loading (**a**) MMT clay (**b**) CNF.

The fracture toughness obtained by the single-edge notched 3-point bending (SEN-3PB) test is shown in Figure 10 and Figure 11. The *K_Ic_* and *G_Ic_* values of the D-clay epoxy nanocomposites are lower than that of the neat epoxy. The addition of 2 wt % clay loading in the D-clay nanocomposites exhibits a toughness of 0.58 MPa∙m^1/2^ and 88.74 J/m^2^ for *K_Ic_* and *G_Ic_*, respectively. This represents a drop of 11.8% for *K_Ic_* and 36.9% for *G_Ic_* as compared to neat epoxy. However, the addition of 2 wt % untreated V-clay exhibits a toughness of 0.834 MPa∙m^1/2^ and 290.1 J/m^2^ for *K_Ic_* and *G_Ic_*, respectively. This is an increase of 23.1% for *K_Ic_* and 104% for *G_Ic_* as compared to neat epoxy.

With CNF, *K_Ic_* and *G_Ic_* values of both D-CNF and V-CNF epoxy nanocomposites are higher than that of the neat epoxy. The addition of 2 wt % clay in the D-CNF nanocomposite exhibits a toughness of 0.878 MPa∙m^1/2^ and 202.5 J/m^2^ for *K_Ic_* and *G_Ic_*, respectively. This is an increase of 33.0% for *K_Ic_* and 43.0% for *G_Ic_* as compared to neat epoxy. The addition of 2 wt % V-CNF exhibits a toughness of 0.836 MPa∙m^1/2^ and 280.4 J/m^2^ for *K_Ic_* and *G_Ic_*, respectively. This represents an increase of 26.7% for *K_Ic_* and 98.0% for *G_Ic_* as compared to neat epoxy.

### 2.6. Fractographic Analysis of D-Clay Nanocomposites

**Figure 12:**
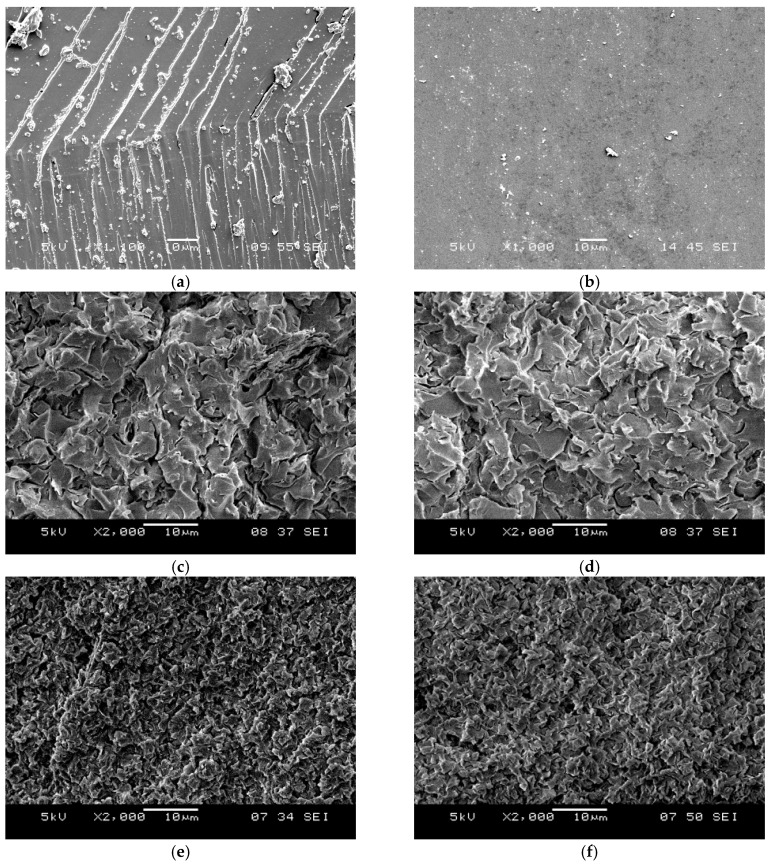
Scanning electron microscopy (SEM) micrograph of different fracture surfaces. (**a**) crack initiation site of neat epoxy; (**b**) fast fracture site of neat epoxy; (**c**) crack initiation site 2 wt % V-clay epoxy; (**d**) fast fracture site of 2 wt % V-clay epoxy; (**e**) crack initiation site of 2 wt % D-clay epoxy; and (**f**) fast fracture site of 2 wt % D-clay epoxy.

**Figure 13:**
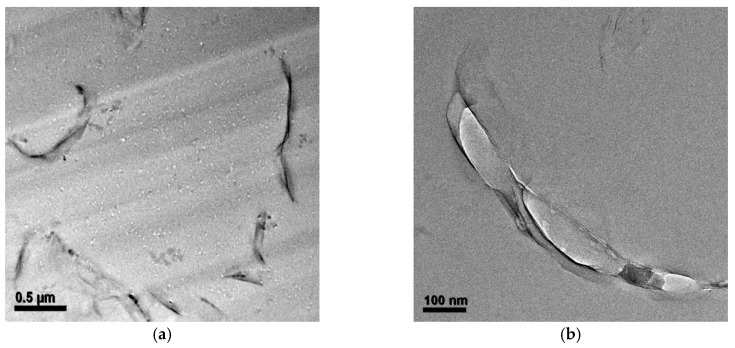

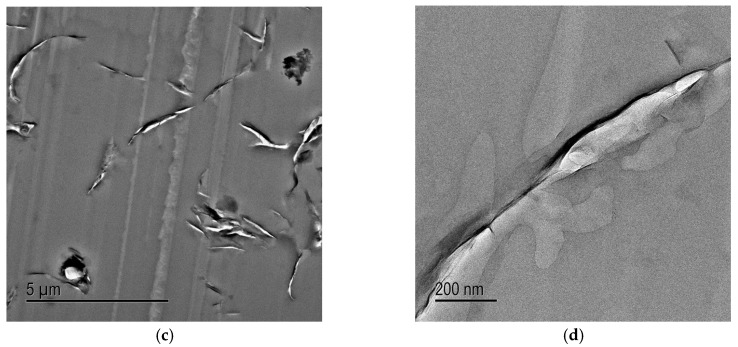
TEM micrographs of sub critical point of the crack initiation site of D-clay (**a**,**b**) and V-clay (**c**,**d**) epoxy composites.

A comparison of neat epoxy, V-clay, and D-clay epoxy composites fracture surfaces are shown in Figure 12. Both composites have rougher surface features compared with neat epoxy, indicating that the crack propagation has occurred by a longer path. The V-clay epoxy nanocomposites have larger facets than D-clay. The larger fractured facets on V-clay composites is probably due to the difference in clay dispersion: a better dispersed D-clay composite (smaller clay unit) is able to provide higher number of clay particles per unit volume. Importantly, there are large numbers of secondary microcracks in the fractured V-clay composites (Figure 13c,d). Micro-cracking releases the crack tip stresses and dissipates additional amount of energy. As a result, the V-clay composites show an enhanced fracture resistance. V-clay does not adhere to the epoxy matrix as strongly as it does in D-clay, which is evidenced by the decrease in Young’s modulus of V-clay composite. Therefore, it is understandable why the secondary cracks can be easily formed during the fracture of V-clay composites.

TEM images were taken at sub-critical points of the crack initiation site of D-clay epoxy nanocomposites (Figure 13a). Several discontinuous cavities are identified in the D-clay layers. Some long microcavities in between clay layers can also be seen. Most of such cavities are formed inside the D-clay layers indicating that delamination of clay layers have indeed taken place. Under high magnification (Figure 13b), these microcracks are confirmed to be initiated within the gallery of clay layers, not at the epoxy D-clay interface. The observation confirms that weakly bonded clay layers delaminated during fracture. This observation that the clay with weak inter-layers strength delaminated during fracture provides a strong support to the earlier explanation for the fracture toughness drop based on fractographic analysis. 

In contrast, in V-clay epoxy composites as the degree of intercalation is poor (Figure 13c) and with little interaction to the matrix, the microcavities (the fracture path) are dominated by the microcracks initiated along the epoxy—V-clay interface (Figure 13d). This is the reason for the observed microcrack deflection and formation of the secondary cracking. These observations again reiterate the importance of surface modification of clay layers in strengthening the interface between the clay and the epoxy matrix. 

In summary, the fracture toughness of the D-clay epoxy nanocomposites is lower than that of the pure epoxy and V-clay epoxy composites. Although the DOPA modification on the clay layers provides very strong adhesion between the clay layers and epoxy matrix, it forces the microcracks to initiate within the clay layers gallery. These weakly bonded clay layers provide an easy path for the fracture to break through. The unmodified V-clay on the other hand, makes it easy to induce secondary cracking. This results in the fracture path to go through a much tortuous path and, thus, increases the fracture toughness of V-clay epoxy composites. We wish to emphasize that the current findings are limited to 2D clay fillers that have the intrinsically weak bonding between its adjacent layers. Such findings are not applicable if a 2D monolayer is used.

### 2.7. Fractographic Analysis of D-CNF Nanocomposites

**Figure 14:**
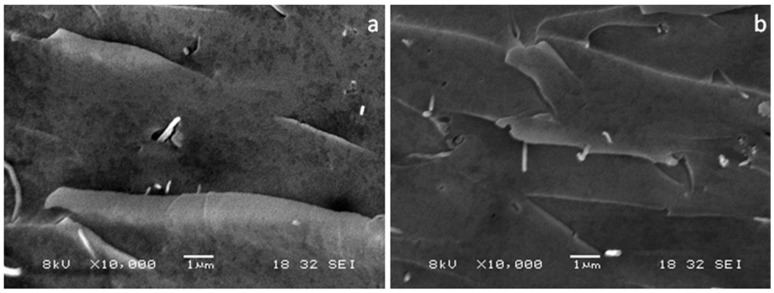

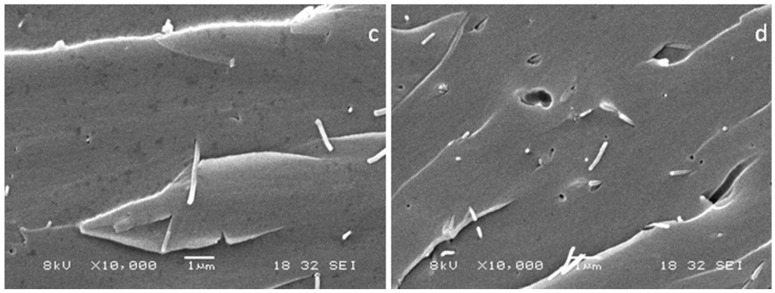
SEM micrograph of different fracture surfaces. (**a**) Crack initiation site 1 wt % D-CNF epoxy; (**b**) fast fracture site of 1 wt % D-CNF epoxy; (**c**) crack initiation site of 1 wt % V-CNF epoxy; and (**d**) fast fracture site of 1 wt % V-CNF epoxy.

**Figure 15:**
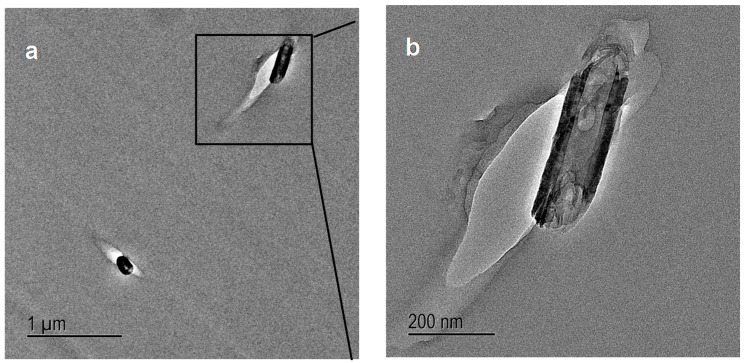
(**a**) TEM micrograph of sub critical point of crack initiation site showing debonding of D-CNF and epoxy; and (**b**) higher magnification of sub critical point of crack initiation site showing debonding of D-CNF and epoxy.

D-CNF and V-CNF epoxy composites have quite similar morphologies on the fractured surface (Figure 15). The V-CNF epoxy composites (Figure 14c,d) generally have larger surface facets than the V-clay epoxy composites (Figure 12c,d). Visual inspection also finds that the fractured surface of V-CNF epoxy composites is smoother than the D-CNF counterpart. This is probably attributed to the stronger ability of the modified fibres in deflecting the running crack because of their better adhesion with the matrix. The unmodified V-CNF, on the other hand, does not adhere to the epoxy matrix as strongly, which has resulted in a decrease in the Young’s modulus as reported earlier. However, despite weaker interface interaction between the fibre and the matrix, which probably accounts for less energy consumption for fibre debonding, the fracture energy (*G_Ic_*) is higher for the V-CNF composite than the D-CNF composite. Crack-tip stress relief and possible formation of secondary cracking could be the main reason for such an enhancement as they are favoured by weak filler-matrix interface as explained in the V-clay case.

The sub-critical point of the crack initiation site of D-CNF nanocomposites is shown in Figure 15. The D-CNFs were dispersed randomly throughout the epoxy matrix. Therefore, the crack is relatively free to propagate through the epoxy matrix before meeting the fibres. Figure 15a shows that microcavities are formed around the D-CNF at the sub-critical point. The matrix around the fibres has clearly deformed and the fibres debonded from the matrix. Such an effect is less pronounced in the case of V-CNF, but nevertheless V-CNF composites are able to compensate through other mechanisms such as stress relief and secondary cracking, as discussed above.

## 3. Materials and Methods 

### 3.1. Materials

The clay used for this experiment was pristine montmorillonite (MMT) clay from Nanocor Inc., (Hoffman Estates, IL, USA), with a cationic exchange capacity of 145 mmol/100 g. MMT has a layered monoclinic crystal structure with a large space between the layers along the c-axis. The mean particle size of NaMMT was calculated to be ~200 μm using the sieve mesh method. The carbon nanofibre (CNF) used was Pyrograf-III (PR-24-XT-PS, Cedarville, OH, USA) with a diameter in the range of 70–150 nm and a length of 50–200 μm, obtained from Applied Science Inc. (Detroit, MI, USA). Tris(hydroxymethyl)aminomethane (TRIS, 99%), dopamaine hydrochloride, 3,4-Di-hydroxyphenethylamine hydrochloride (DOPA, 98%) and acetone (Tech grade) were obtained from Sigma-Aldrich (Singapore). Epoxy precursor DER 332 was supplied by Dow Chemicals (Singapore) and amine hardener Ethacure-100LC was supplied by Albemarle Corp (Baton Rouge, LA, USA). All chemicals were used as received.

### 3.2. D-Clay and D-CNF Preparation

One gram of MMT clay was added into 100 mL of deionized water and mechanically stirred for 24 h. This was followed by resting it under ambient condition for another 24 h. After that, the settlement at the bottom of the beaker was removed. The remaining suspension was added into 250 mL 10 nM TRIS buffer solution and mechanically stirred for 15 min to achieve a homogenous clay dispersion in the new solution. Different amounts of DOPA, corresponding to 0.1 to 2.0 mg/mL solution concentration, was then added and continuously stirred for 2 h at ambient condition. The thermal stability was determined by the starting temperature of degradation using TGA. It was found that 1.5 mg/mL was able to provide the most stable DOPA coating and sufficient coating thickness, and the fillers were well dispersed. Therefore, this concentration was used for all the experiments unless otherwise stated. The suspension was then centrifuged at 7500 rpm for 20 min. This step of wash-centrifuge was repeated for four times to remove the unattached DOPA oligomers. A dark slurry-like solution was obtained after the washing. Finally, D-clay was produced by drying the solution in a vacuum oven for 24 h at 70 °C. In the same way, D-CNF slurry was prepared.

### 3.3. Characterisation of D-Clay

X-ray photoelectron spectroscopy (XPS) measurement was carried out using a Kratos Analytical AXIS HIS spectrometer (Shimadzu Corporation, Kyoto, Japan) with a monochromatized Al Ka X-ray source (1486.6 eV photons) to verify the polydopamine coating on the clay. Thermogravimetric analysis (TGA) was carried out using a TGA Q2950 (TA Instruments, New Castle, DE, USA) under an atmosphere environment where the samples were heated from 25 °C to 140 °C and kept isothermally for 40 min before being further heated at a rate of 10 °C/min to 850 °C. Fourier transform infrared spectroscopy (FTIR) was carried out using a Perkin-Elmer 2000 spectrometer (Perkin-Elmer, Waltham, MA, USA) scanning in the range of 400 cm^−1^ to 4000 cm^−1^ with a resolution of 4 cm^−1^. Crystal structure was determined using X-ray diffraction (XRD) on a PANalytical X’Pert PRO X-ray diffractometer (Almelo, The Netherlands) with Cu K radiation (λ = 0.154 nm). D-clay was pounded and grinded into powder form before the XRD measurement. Samples were scanned with a scanning rate of 2°/min from 2° to 35° in ambience. The average D-spacing was then calculated using the Bragg’s law based on the measured diffraction angle.

### 3.4. Preparation of Epoxy Nanocomposites

To prepare the D-clay and D-CNF epoxy nanocomposites, precursor DER 332 was introduced into the slurry and the solution was vigorously stirred for 2 h using a homogeniser, after which acetone was removed using a rotary evaporator and the remaining slurry mixture was dried in a vacuum oven at 85 °C for 48 h. The amine hardener Ethacure-100LC was then added to the dried epoxy at the epoxy/amine hardener ratio of 3.8:1 (*w*:*w*). The mixture was then stirred using a mechanical stirrer for 30 min to achieve a homogenous mixture of the epoxy and amine hardener. The mixture was then degassed at 50 °C and cured using a curing profile of 100 °C for 2 h, then 180 °C for 5 h.

### 3.5. Mechanical Property Measurement of Nanocomposites

The storage moduli of the samples were measured using a DMA 2980 dynamic mechanical analyser (TA Instruments, New Castle, DE, USA) in the single cantilever mode. The samples were moulded into a rectangular size of 30 mm × 10 mm × 3 mm and scanned from 25 °C to 250 °C at a heating rate of 3 °C/min using a frequency of 1 Hz. 

Young’s modulus of the nanocomposites was determined on an Instron 5567 Universal Tester (Norwood, MA, USA) at a cross-head speed of 1 mm/min according to ASTM D-5083-10 test standard. The length and thickness of the tested portion is 50 mm × 3 mm, and the total length is 80 mm. The strain was recorded using a strain gauge at a gauge length of 25 mm. 

Fracture toughness was measured using the SEN-3PN test to obtain the Mode-I critical stress intensity factor (*K_Ic_*). The dimensions of the specimens are 60 mm × 12.7 mm × 4.0 mm, and the span is 50 mm. A sharp notch was introduced to the specimen by tapping a hammer on a razor blade inserted in to the sample. At least five valid results were obtained for each condition. The critical strain energy release rate (*G_Ic_*) was calculated from the *K_Ic_*, assuming a Poisson’s ratio of 0.35.

### 3.6. Electron Microscopic Observation

A scanning electron microscope (SEM) was used to examine the fracture surface of the tested SEN-3PB specimens. The samples were coated with a thin layer of gold and observed using a JEOL 6700 SEM (JOEL, Tokyo, Japan). The accelerating voltage was 5 kV. To study the microstructure at the crack tip damage zone, the area in front of the crack tip was located and carefully trimmed down manually with a sharp razor blade to the size suitable for ultra-microtoming. Subsequently, the face of the sample was trimmed by a freshly-made glass knife in the Leica Ultracut Microtome. Finally, the sample was carefully sectioned to 50–70 nm thick sections at a rate of 0.3 mm/s using a diamond knife in the same microtome machine at ambient temperature. The thin sections were then collected by a water-loop onto a carbon-coated copper grid. These sections were observed under a JOEL 2100 transmission electron microscope (TEM, JOEL, Tokyo, Japan) at 200 kV in bright field mode.

## 4. Conclusions

The effects of PDOPA-coated MMT clay, which has a weak bonding between adjacent layers, and carbon nanofibre as fillers on the mechanical properties of their epoxy nanocomposites are evaluated and compared with the unmodified counterparts. The storage modulus is enhanced at very low filler loadings. Young’s modulus of D-clay and D-CNF nanocomposites are enhanced while the unmodified counterparts display a reduction. The decrease in the Young’s modulus of the untreated filler composite proves the importance of filler-matrix adhesion in effective stress transfer when the elasticity constant is concerned. 

The effects of DOPA surface treatment and filler shape on the fracture properties of epoxy nanocomposites, however, prove to be rather more complex. The *K_Ic_* and *G_Ic_* values of the D-clay epoxy nanocomposites are lower than that of the neat epoxy and that of V-clay composites. Through fractographic analysis and crack tip clay-epoxy interaction analysis, we were able to conclude that the strong adhesion of the clay surface with matrix forces the clay layers to delaminate under crack tip stresses. Since the delamination toughness of the clay layers is much lower, the low energy consumption of clay delamination and the strong adhesion between clay and the matrix lead to a lower toughness of the composites. On the other hand, virgin clay composites possess increased fracture toughness mainly due to the formation of secondary cracking induced by the weak interface between the clay and the epoxy matrix.

The fracture toughness increases with both modified and unmodified one-dimensional CNF nanocomposites. Through fracture surface and crack tip damage investigation using electron microscopy, it was found that the 1D filler possesses more versatile means to enhance the fracture resistance regardless of the strength of filler-matrix interface. For the brittle epoxy matrix, the current study suggests that both the form factor and the intrinsic properties of the nanofillers have strong effects on the fracture energy of the epoxy composites. The outcome of this study could serve as a guideline for engineering the mechanical properties of future nanocomposites using a brittle matrix.
